# Construction and validation of a nomogram for predicting overall survival of patients with stage III/IV early−onset colorectal cancer

**DOI:** 10.3389/fonc.2024.1332499

**Published:** 2024-04-10

**Authors:** Wanbin Yin, Wenju Pei, Tao Yu, Qi Zhang, Shiyao Zhang, Maorun Zhang, Gang Liu

**Affiliations:** ^1^ Department of General Surgery, Tianjin Medical University General Hospital, Tianjin, China; ^2^ Department of Anorectal Surgery, Affiliated Hospital of Jining Medical University, Jining, China; ^3^ Department of Oncology, Tianjin Medical University General Hospital, Tianjin, China

**Keywords:** early-onset, colorectal cancer, overall survivals, prognostic factors, nomogram

## Abstract

**Purpose:**

This study aimed to identify prognostic factors and develop a nomogram for predicting overall survival (OS) in stage III/IV early-onset colorectal cancer (EO-CRC).

**Methods:**

Stage III/IV EO-CRC patients were identified from the Surveillance, Epidemiology, and End Results (SEER) database between 2010 and 2015. The datasets were randomly divided (2:1) into training and validation sets. A nomogram predicting OS was developed based on the prognostic factors identified by Cox regression analysis in the training cohort. Moreover, the predictive performance of the nomogram was assessed using the receiver operating characteristic (ROC) curves, calibration plots, and decision curve analysis (DCA). Subsequently, the internal validation was performed using the validation cohort. Finally, a risk stratification system was established based on the constructed nomogram.

**Results:**

Of the 10,387 patients diagnosed with stage III/IV EO-CRC between 2010 and 2015 in the SEER database, 8,130 patients were included. In the training cohort (n=3,071), sex, marital status, race/ethnicity, primary site, histologic subtypes, grade, T stage, and N stage were identified as independent prognostic variables for OS. The 1-, 3-, and 5-year area under the curve (AUC) values of the nomogram were robust in both the training (0.751, 0.739, and 0.723) and validation cohorts (0.748, 0.733, and 0.720). ROC, calibration plots, and DCA indicated good predictive performance of the nomogram in both the training and validation sets. Furthermore, patients were categorized into low-, middle-, and high-risk groups based on the nomogram risk score. Kaplan-Meier curve showed significant survival differences between the three groups.

**Conclusion:**

We developed a prognostic nomogram and risk stratification system for stage III/IV EO-CRC, which may facilitate clinical decision-making and individual prognosis prediction.

## Introduction

Colorectal cancer (CRC) is the second most deadly and third most common cancer worldwide (1). While CRC incidence has decreased in individuals ≥50 years of age, it has increased globally in individuals younger than 50 years in the past decades, which has been defined as early-onset CRC (EO-CRC) (2–7). EO-CRC has been in the spotlight recently, which would account for about 11% of colon cancers and 23% of rectal cancers in 2030 (8). Unfortunately, the reasons of this increase in EO-CRC remain unclear and probably multifactorial (9). Additionally, clinical features of EO-CRC, often diagnosed with advanced stage disease (6, 10–12), differ from those of later-onset disease (3, 4). Overall, EO-CRC contributes significantly to the global cancer burden. Hence, to facilitate clinical decision-making, it is important to predict the prognosis of EO-CRC patients.

Although widely used to examine the survival of CRC, the American Joint Committee on Cancer (AJCC) staging system is far from perfect. Several studies have identified prognostic risk factors for EO-CRC and developed the prognostic nomograms. However, some problems remain. First, flow chat was not available in two studies (13, 14). Second, three nomograms (15–17) were associated with too many prognosis factors (≥ 12), which reduced its practicability. Last, and most importantly, several nomograms were unreasonable in clinical context. For example, it was paradoxical that patients with grade I had a worse prognosis than those with grade II (15, 16, 18). Similar to the “grade paradox”, there were also “race/ethnicity paradox” (13, 14, 19, 20), “T stage paradox” (16, 18, 19), “histologic subtypes paradox” (21), and “primary site paradox” (15, 18, 21, 22). Thus, more high‐quality research is urgently needed.

To the best of our knowledge, to date, no nomogram has been constructed to predict the prognosis of patients with stage III/IV EO-CRC. Therefore, this study aimed to develop and validate a prognostic nomogram predicting overall survival (OS) for stage III/IV EO-CRC based on the Surveillance, Epidemiology, and End Results (SEER) database.

## Methods

### Patient selection

The clinicopathological data of patients with EO-CRC between 2010 and 2015 were extracted using SEER*Stat 8.4.1 software. Primary tumor sites were divided into anatomical subsites: cecum (C18.0), ascending colon (C18.2), hepatic flexure of colon (C18.3), transverse colon (C18.4), splenic flexure of colon (C18.5), descending colon (C18.6), sigmoid colon (C18.7), rectosigmoid junction (C19.9), and rectum (C20.9). Based on the third edition of the International Classification of Diseases Oncology Special Edition, the histologic subtypes included adenocarcinoma (8140/3, 8144/3, 8201/3,8210/3, 8211/3, 8213/3, 8220/3, 8221/3, 8255/3,8260/3, 8261/3, 8262/3, 8263/3, 8310/3, 8323/3), mucinous adenocarcinoma (MA) (8480/3, 8481/3), and signet ring cell carcinoma (SRCC) (8490/3).

The inclusion criteria were patients diagnosed with stage III/IV EO-CRC (pathologically confirmed) between 2010 and 2015. Exclusion criteria included patients younger than 18 years, and those lacking complete clinicopathological and survival information.

### Data collection

The collected variables included age, sex, marital status, race/ethnicity, primary site, histologic subtypes, grade, AJCC stage, T stage, N stage, survival time, and vital status record. Primary sites comprise right-sided colon (cecum, ascending colon, hepatic flexure, and transverse colon), left-sided colon (splenic flexure, descending colon, sigmoid colon, and rectosigmoid junction), and rectum (23). Race/ethnicity was divided into five categories: Hispanic, Non-Hispanic White (NHW), Non-Hispanic Black (NHB), Non-Hispanic Asian or Pacific Islander (NHAPI), and Non-Hispanic American Indian/Alaska Native (NHAIAN). The tumor-node-metastasis (TNM) stage was determined according to the AJCC seventh edition criteria. OS is defined as the time from diagnosis to death from any cause or the time of the last follow-up.

### Development of the prognostic nomogram

The datasets were randomly divided (2:1) into the training and validation sets. In the training cohort, univariate and multivariate Cox regression analyses were performed to identify the prognostic factors of patients with stage III/IV EO-CRC. The independent risk factors were used to construct the prognostic nomogram.

### Validation of the prognostic nomogram

The discrimination ability of the prognostic nomogram was examined by the receiver operating characteristic (ROC) curves and the area under the curves (AUC). The calibration ability was evaluated by the calibration plot. Additionally, decision curve analysis (DCA) was performed to assess clinical utility by quantifying the net benefits at different threshold probabilities.

### Risk stratification

Based on the constructed nomogram, the total risk score was calculated for each patient. X-tile software (version 3.6.1, Yale University) was used to identify the optimal cutoff values for the total risk score. According to the cutoff values of risk score, patients were classified into low-, middle-, and high-risk groups. Kaplan–Meier curves and the log-rank test were used to compare the survival differences between different groups.

### Statistical analysis

R software (version 4.2.1, https://www.r-project.org) and relevant packages were used to construct and validate the prognostic nomogram. A p-value < 0.05 was considered significant.

## Results

### Patient characteristics

**Figure 1:**
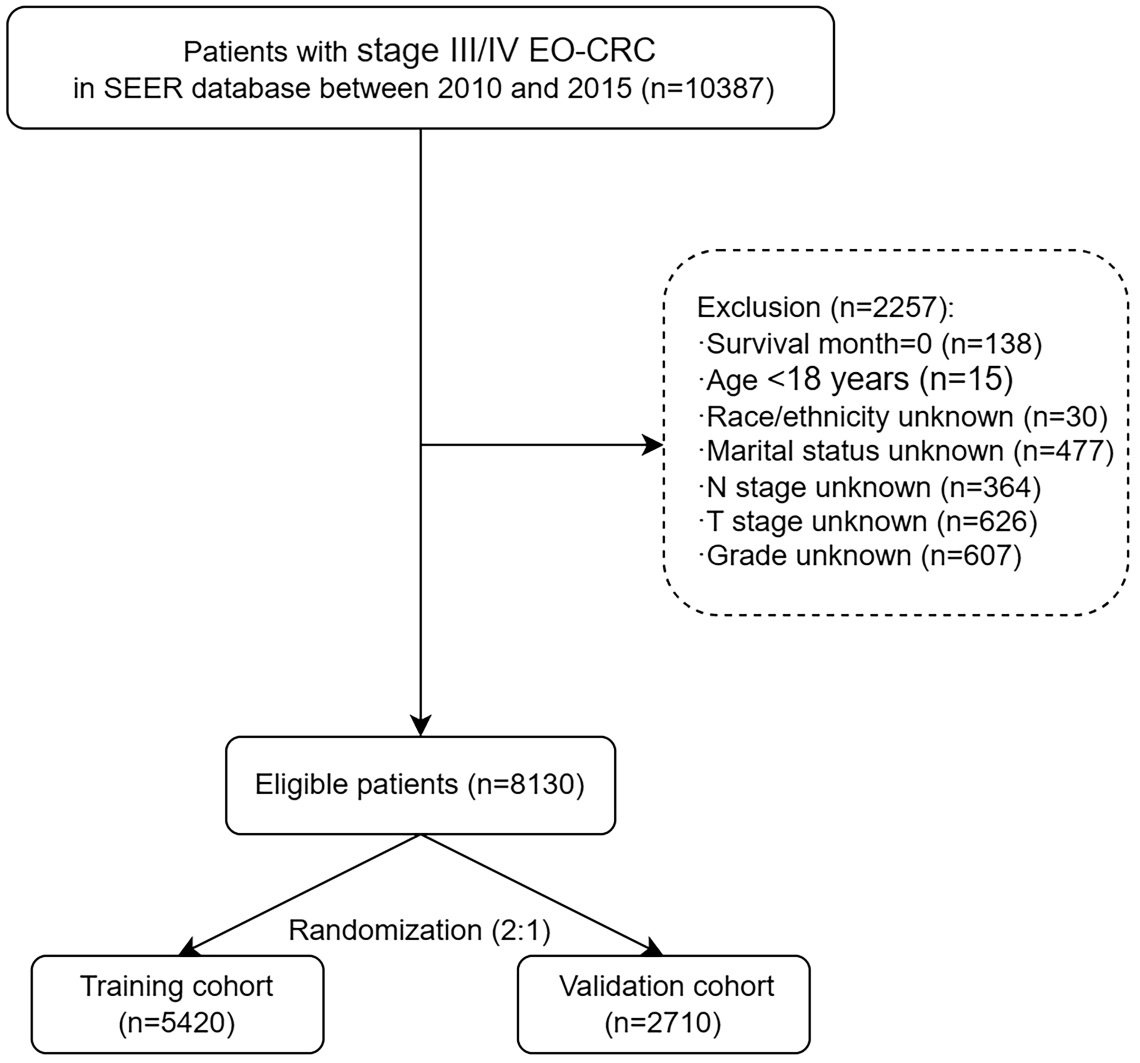
Flow chart of study screening.

Overall, 10,387 patients with stage III/IV EO-CRC were identified between 2010 and 2015 from the SEER database. According to the inclusion and exclusion criteria, 8,130 patients with stage III/IV EO-CRC were included in the final analysis. The study screening flow chart is shown in **Figure 1**. Subsequently, the datasets were randomly divided (2:1) into a training set (n=3071) and a validation set (n=1535), with no statistical difference. The demographic and clinicopathological characteristics of stage III/IV EO-CRC patients are summarized in **Table 1**. The baseline characteristics of patients stratified by AJCC stage are shown in **Supplementary Table S1**.

**Table 1:** Demographic and clinical characteristics of patients with stage III/IV EO-CRC.

Variables	Total	Training set	Validation set	P value
	(n=8130)	(n=5420)	(n=2710)	
Age (years)	42.17 ± 6.20	42.17 ± 6.2	42.16 ± 6.21	0.891
Sex				0.894
Female	3757 (46.2%)	2508 (46.3%)	1249 (46.1%)	
Male	4373 (53.8%)	2912 (53.7%)	1461 (53.9%)	
Marital status				0.930
Married	4718 (58.0%)	3143 (58%)	1575 (58.1%)	
SDW	3412 (42.0%)	2277 (42%)	1135 (41.9%)	
Race/ethnicity				0.657
Hispanic	1430 (17.6%)	952 (17.6%)	478 (17.6%)	
NHW	4670 (57.4%)	3129 (57.7%)	1541 (56.9%)	
NHB	1119 (13.8%)	743 (13.7%)	376 (13.9%)	
NHAPI	830 (10.2)	548 (10.1%)	282 (10.4%)	
NHAIAN	81 (1.0%)	48 (0.9%)	33 (1.2%)	
Primary site				0.228
Right colon	2087 (25.7%)	1368 (25.2%)	719 (26.5%)	
Left colon	3736 (46.0%)	2526 (46.6%)	1210 (44.6%)	
Rectum	2307 (28.4%)	1526 (28.2%)	781 (28.8%)	
Histologic subtypes				0.154
Adenocarcinoma	7351 (90.4%)	4919 (90.8%)	2432 (89.7%)	
MA/SRCC	779 (9.6%)	501 (9.2%)	278 (10.3%)	
Grade				0.175
Grade I	412 (5.1%)	262 (4.8%)	150 (5.5%)	
Grade II	5724 (70.4%)	3857 (71.2%)	1867 (68.9%)	
Grade III	1672 (20.6%)	1093 (20.2%)	579 (21.4%)	
Grade IV	322 (4.0%)	208 (3.8%)	114 (4.2%)	
AJCC stage				0.519
III	5197 (63.9%)	3451 (63.7%)	1746 (64.4%)	
IV	2933 (36.1%)	1969 (36.3%)	964 (35.6%)	
T stage				0.571
T1	492 (6.1%)	342 (6.3%)	150 (5.5%)	
T2	543 (6.7%)	358 (6.6%)	185 (6.8%)	
T3	4981 (61.3%)	3316 (61.2%)	1665 (61.4%)	
T4	2114 (26.0%)	1404 (25.9%)	710 (26.2%)	
N stage				0.807
N0	588 (7.2%)	399 (7.4%)	189 (7%)	
N1	4535 (55.8%)	3016 (55.6%)	1519 (56.1%)	
N2	3007 (37.0%)	2005 (37%)	1002 (37%)	

EO-CRC, early-onset colorectal cancer; SDW, separated, single, divorced, domestic partner or unmarried, widowed; NHW, Non-Hispanic White; NHB, Non-Hispanic Black; NHAPI, Non-Hispanic Asian or Pacific Islander; NHAIAN, Non-Hispanic American Indian/Alaska Native; MA, mucinous adenocarcinoma; SRCC, signet ring cell carcinoma; AJCC, American joint committee on cancer; T, Tumor; N, Node.

### Development of the prognostic nomogram

**Figure 2:**
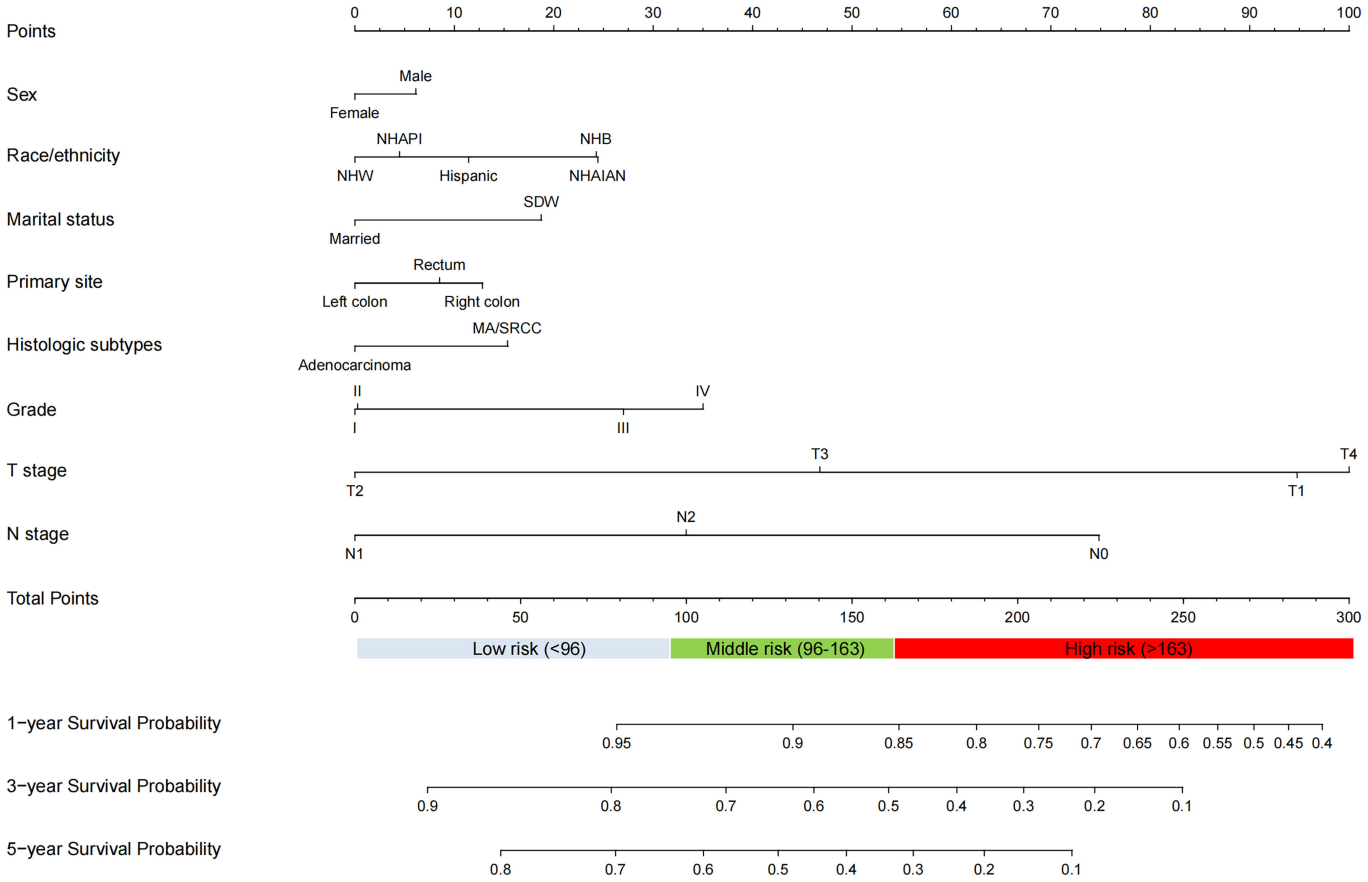
Nomogram for predicting 1-, 3-, and 5-year OS for patients with stage III/IV EO-CRC.

The univariate and multivariate Cox regression analysis revealed that sex, marital status, race/ethnicity, primary site, histologic subtypes, grade, T stage, and N stage were identified as independent prognostic factors for OS in the training cohort (**Table 2**). Accordingly, these independent prognostic factors were utilized to construct the nomogram for predicting the 1-, 3-, and 5-year OS of stage III/IV EO-CRC patients (**Figure 2**).

**Table 2:** Univariate and multivariate analysis of OS in the training cohort.

Variables	Univariate analysis		Multivariate analysis	
	HR (95% CI)	P value	HR (95% CI)	P value
Age (years)	1.00 (0.99-1.00)	0.599		
Sex				
Female	Reference		Reference	
Male	1.10 (1.02-1.20)	0.018	1.09 (1.00-1.18)	0.046
Marital status				
Married	Reference		Reference	
SDW	1.39 (1.28-1.50)	<0.001	1.29 (1.19-1.40)	<0.001
Race/ethnicity				
Hispanic	Reference		Reference	
NHW	0.85 (0.76-0.95)	0.004	0.86 (0.77-0.96)	0.006
NHB	1.26 (1.10-1.45)	<0.001	1.19 (1.04-1.37)	0.013
NHAPI	0.91 (0.78-1.07)	0.262	0.91 (0.78-1.07)	0.251
NHAIAN	1.44 (0.98-2.13)	0.064	1.19 (0.81-1.76)	0.38
Primary site				
Left colon	Reference		Reference	
Right colon	1.35 (1.22-1.48)	<0.001	1.20 (1.08-1.32)	<0.001
Rectum	0.92 (0.83-1.01)	0.088	1.19 (1.08-1.31)	0.001
Histologic subtypes				
Adenocarcinoma	Reference		Reference	
MA/SRCC	1.54 (1.36-1.74)	<0.001	1.23 (1.08-1.40)	0.002
Grade				
Grade I	Reference		Reference	
Grade II	1.04 (0.85-1.28)	0.675	1.00 (0.82-1.23)	0.972
Grade III	1.80 (1.46-2.23)	<0.001	1.44 (1.16-1.79)	0.001
Grade IV	2.09 (1.60-2.72)	<0.001	1.61 (1.23-2.10)	0.001
T stage				
T1	Reference		Reference	
T2	0.25 (0.19-0.33)	<0.001	0.28 (0.21-0.36)	<0.001
T3	0.52 (0.44-0.60)	<0.001	0.52 (0.45-0.61)	<0.001
T4	1.24 (1.06-1.45)	0.007	1.07 (0.91-1.26)	0.399
N stage				
N0	Reference		Reference	
N1	0.30 (0.27-0.34)	<0.001	0.36 (0.32-0.42)	<0.001
N2	0.54 (0.48-0.62)	<0.001	0.57 (0.50-0.65)	<0.001

EO-CRC, early-onset colorectal cancer; SDW, separated, single, divorced, domestic partner or unmarried, widowed; NHW, Non-Hispanic White; NHB, Non-Hispanic Black; NHAPI, Non-Hispanic Asian or Pacific Islander; NHAIAN, Non-Hispanic American Indian/Alaska Native; MA, mucinous adenocarcinoma; SRCC, signet ring cell carcinoma; T, Tumor; N, Node.

### Validation of the prognostic nomogram

**Figure 3:**
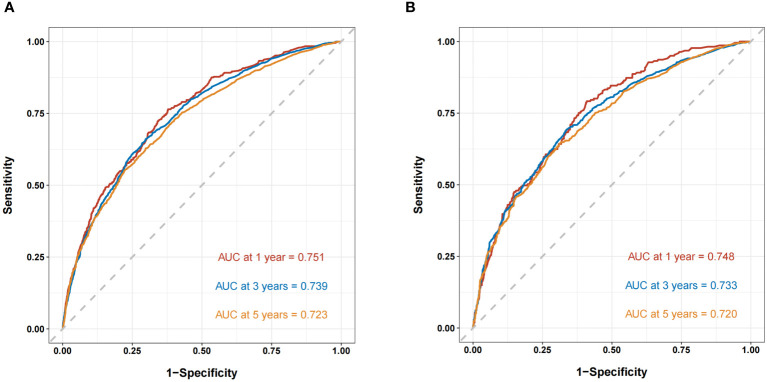
The ROC curves of the nomogram predicting OS in the training cohort **(A)** and validation cohort **(B)**.

**Figure 4:**
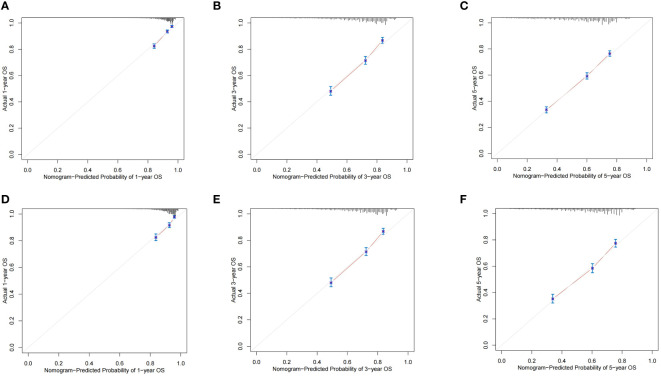
The calibration plots of the nomogram predicting 1-,3- and 5-year OS in the training cohort **(A–C)** and validation cohort **(D–F)**.

**Figure 5:**
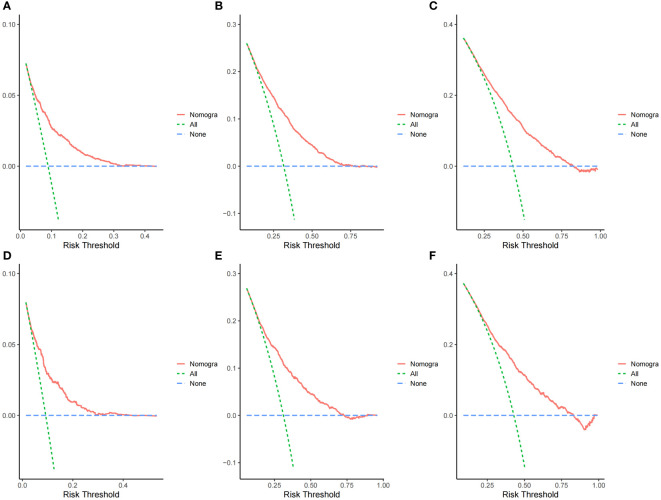
Decision curve analysis curves of the nomogram predicting 1-,3- and 5-year OS in the training cohort **(A–C)** and validation cohort **(D–F)**.

The ROC curves demonstrated that the discriminating ability of the prediction model was robust both in the training and validation sets. The AUC values of the nomogram predicting 1-, 3-, and 5-year OS in the training cohort were 0.751, 0.739, and 0.723, respectively. Similarly, in the validation cohort, the corresponding values were 0.748, 0.733, and 0.720, respectively (**Figure 3**). Additionally, the calibration plots demonstrated the prognostic nomogram’s strong calibration capability (**Figure 4**). Furthermore, the DCA curves suggested good clinical utility (**Figure 5**).

### Risk stratification

**Figure 6:**
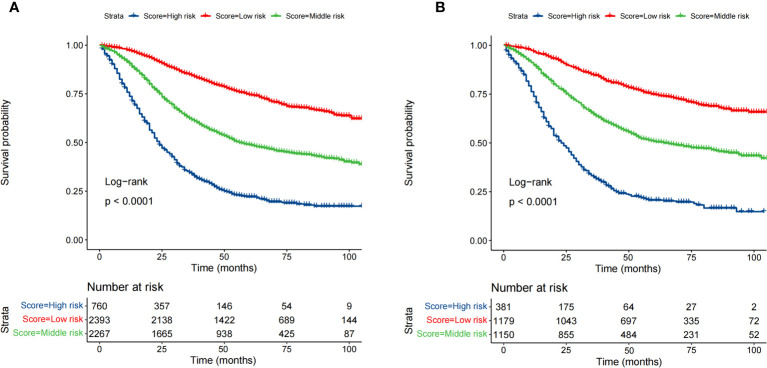
Survival analysis of the training cohort **(A)** and the validation cohort **(B)** by the risk score calculated by the nomogram.

Based on the optimal cutoff values for the total risk score, patients in the training cohort were grouped into the low-risk (≤95.5), middle-risk (95.5-162.5), and high-risk groups (>162.5) (**Supplementary Figure S1**). Similarly, patients in the validation cohort were categorized into low-risk (≤96), middle-risk (96-162.5), and high-risk groups (>162.5) (**Supplementary Figure S2**). Kaplan–Meier curves, using the log-rank test, revealed significant differences in OS among the three risk subgroups in both the training and validation cohorts (**Figure 6**).

## Discussion

Based on the SEER database, 8,130 stage III/IV EO-CRC patients were included. The present study identified that eight variables, including sex, marital status, race/ethnicity, primary site, histologic subtypes, grade, T stage, and N stage, were independent predictors for OS of patients with stage III/IV EO-CRC. Utilizing these variables, a nomogram with favorable performance predicting 1-, 3-, and 5-year OS was developed and validated. Furthermore, a risk stratification was successfully established based on the total risk score determined by the nomogram.

The initial nomogram demonstrated that AJCC stage had the most substantial impact on OS of the patients with stage III/IV EO-CRC. Based on our previous nomogram, risk score for stage III and IV is 0 and 100, respectively. This limits its clinical application. Therefore, AJCC stage was not included when constructing the new nomogram. Our new nomogram demonstrated that T stage contributed the most to prognosis, followed by N stage, grade, race, marital status, histologic subtypes, primary site, and sex. All eight prognostic variables are readily available and clinically reasonable. Our findings suggest that right colon cancer had a worse OS than rectum cancer, and rectum cancer had a worse OS than left colon cancer in stage III/IV EO-CRC. These results are in accord with published studies (16, 22).

Consistent with the literature (13, 15, 16, 18–20, 24), this study showed that age was not a significant prognostic factor for the survival of patients with stage III/IV EO-CRC. However, a few studies have presented contrasting views. A survival nomogram for stage I–III EO-CRC patients revealed that patients with older age had a worse survival than those with younger age (14). In contrast, another nomogram for combined lymphatic metastases in EO-CRC patients demonstrated that patients with younger age had a worse survival than those with older age (21). These divergent findings necessitate cautious interpretation.

Our study demonstrated that sex had a minor influence on prognosis. These results are in accord with three studies (20, 22, 25) indicating that male EO-CRC patients exhibited slightly poorer survival compared to their female counterparts. However, several studies (13, 15, 16, 18, 19, 24) indicated that sex was not a significant prognostic factor for the survival of EO-CRC patients. Collectively, the influence of sex on the survival of EO-CRC patients warrants further investigation.

In the present study, race/ethnicity was divided into five categories: Hispanic, NHW, NHB, NHAPI, and NHAIAN. Our nomogram showed that NHW patients had the best prognosis in stage III/IV EO-CRC, followed by NHAPI, Hispanic, NHB, and NHAIAN patients. A study using data from the SEER database demonstrated that the cancer-specific survival at five years for NHW, NHAPI, Hipanic, NHAIAN, and NHB patients with EO-CRC was 70.11, 68.70, 68.31, 65.67, and 65.58 months, respectively (26). These results were almost the same as ours. Additionally, many studies have investigated the racial/ethnic disparities in survival among EO-CRC patients. While the criteria for racial/ethnic classification may vary, the findings of these studies consistently indicate that NHB patients have the poorest prognosis, whereas NHW patients exhibit the most favorable prognosis (27–32). Our results also support this view.

Generally speaking, a higher T stage means a worse prognosis (13, 14, 21). Surprisingly, our study indicated that, compared to T1 patients, those with T2 (adjusted hazard ratio [aHR], 0.28; 95% confidence interval [CI], 0.21-0.36), T3 (aHR, 0.52; 95% CI, 0.45-0.61) and > T4 (aHR, 1.07; 95% CI, 0.91-1.26) had better OS independent of other variables. This seems a rather paradoxical finding. Nevertheless, we believe this to be an important clinical finding rather than a paradox. The most possible reason is that advanced tumors with light intestinal wall invasion may represent a biologically aggressive phenotype. More studies are needed to elucidate the underlying mechanism.

Similar to T stage, a higher N stage means a worse prognosis (14, 20, 24). In contrast, our study suggested another paradoxical finding. Compared to N0 patients, those with N1 (aHR, 0.36; 95% CI, 0.32-0.42), and N2 (aHR, 0.57; 95% CI, 0.50-0.65) had a better prognosis. The participants of our study were patients with stage III/IV EO-CRC. Therefore, the patients either present with lymph node metastasis or distant metastasis. Patients with N1 and N2 may not have distant metastasis, while patients with N0 must have distant metastasis. Thus, although paradoxical, this finding is logical in stage III/IV EO-CRC.

A higher tumor grade is commonly associated with a poorer prognosis, as confirmed in our study. However, a nomogram predicting OS for metastatic EO-CRC revealed counterintuitive trends: grade I patients had a worse prognosis than grade II, and grade III worse than grade IV (15). Clearly, these results are illogical. Based on their nomogram, the risk scores for grade I, II, III, and IV were 20, 0, 81, and 60, respectively. In the training cohort, the sample size for patients with grade I, II, III, and IV were 72, 1212, 378, and 67, respectively. We speculate that the small sample size for patients with grade I and IV (<100) may lead to the “grade paradox”. Notably, several other studies were also limited by “grade paradox” (16, 18, 21). We speculated that this survival paradox resulted from the small sample sizes.

There are several strengths to the present study. First, to the best of our knowledge, this is the first predictive nomogram focusing on patients with stage III/IV EO-CRC. Second, the AUC values of the nomogram predicting 1-, 3-, and 5-year OS were all greater than 0.71. The calibration plots showed a good calibration ability and the DCA curves indicated a good clinical utility. Moreover, the internal validation also demonstrated satisfactory results. Third, of the 10,387 patients with stage III/IV EO-CRC, only 2257 patients (lacking essential clinical and survival information) were excluded. Thus, 8,130 patients were included in the final analysis, suggesting a strong representativeness. Fourth, based on the nomogram, a risk stratification system was successfully established to identify high risk patients.

Of course, some limitations must be recognized in the present study. First, it was a retrospective study, limiting the generalizability of the results. In the future, prospective studies are warranted to verify the findings. Second, there was no detailed information on CEA, radiotherapy, chemotherapy, and surgical treatment in the SEER database. Third, the SEER database did not include potential prognostic factors for EO-CRC, such as systemic immune inflammation index (33), geriatric nutrition risk index (33), symptom duration of 3 months or more (34), carbohydrate antigen 19-9 (35), and cell cycle-related genes (36). Fourth, our data was exclusively from the SEER database, representing only a portion of the US population, potentially limiting the applicability of our results to other regions or countries. Last, it should be stated that although the internal validation demonstrated satisfactory results, external validation was not performed. This problem will be addressed in our future studies.

Patients with EO-CRC differ from those with later-onset CRC in underlying molecular mechanisms (37–40), clinical features, and treatment (41). This special population warrants further attention in the future.

## Conclusions

Independent risk factors for OS in stage III/IV EO-CRC patients included sex, marital status, race/ethnicity, primary site, histologic subtypes, grade, T stage, and N stage. An effective nomogram and risk stratification system were established, potentially enhancing clinical decision-making and individual prognosis prediction.

## Data availability statement

The datasets presented in this study can be found in online repositories. The names of the repository/repositories and accession number(s) can be found below: The data sets analyzed in this study are available in the SEER database.

## Ethics statement

The requirement of ethical approval was waived by Tianjin Medical University General Hospital for the studies involving humans because de-identified data were used in this study. The studies were conducted in accordance with the local legislation and institutional requirements. The ethics committee/ institutional review board also waived the requirement of written informed consent for participation from the participants or the participants' legal guardians/next of kin because of the retrospective nature of the study.

## Author contributions

WY: Conceptualization, Data curation, Formal analysis, Methodology, Writing – original draft. WP: Conceptualization, Data curation, Formal analysis, Methodology, Writing – original draft. TY: Methodology, Software, Visualization, Writing – review & editing. QZ: Methodology, Software, Visualization, Writing – review & editing. SZ: Data curation, Validation, Writing – review & editing. MZ: Data curation, Validation, Writing – review & editing. GL: Conceptualization, Funding acquisition, Resources, Supervision, Writing – review & editing.

## Supplementary material

Supplementary Figure 1 Identification of the best cut-off point of total risk score in the training cohort through X-tile software.

Supplementary Figure 2 Identification of the best cut-off point of total risk score in the validation cohort through X-tile software.
