# Supplementary figures and images for: Construction and validation of a nomogram for predicting overall survival of patients with stage III/IV early−onset colorectal cancer
Source: Front Oncol. 2024 Apr 10;14:1332499. doi: 10.3389/fonc.2024.1332499 (PMC11040690; doi:10.3389/fonc.2024.1332499)

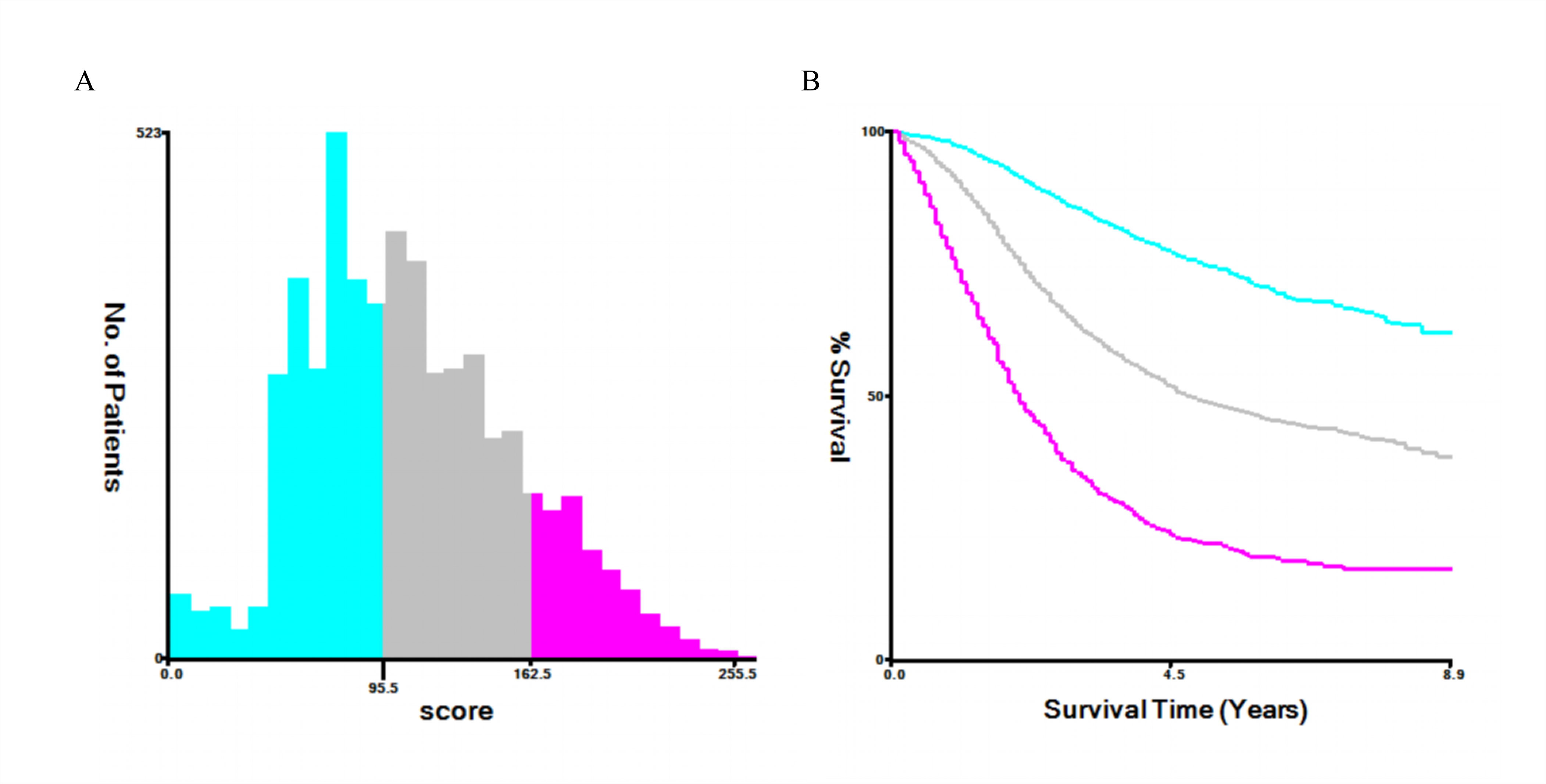

Supplement: Supplementary Figure 1 — Identification of the best cut-off point of total risk score in the training cohort through X-tile software. [file Image_1.tif]

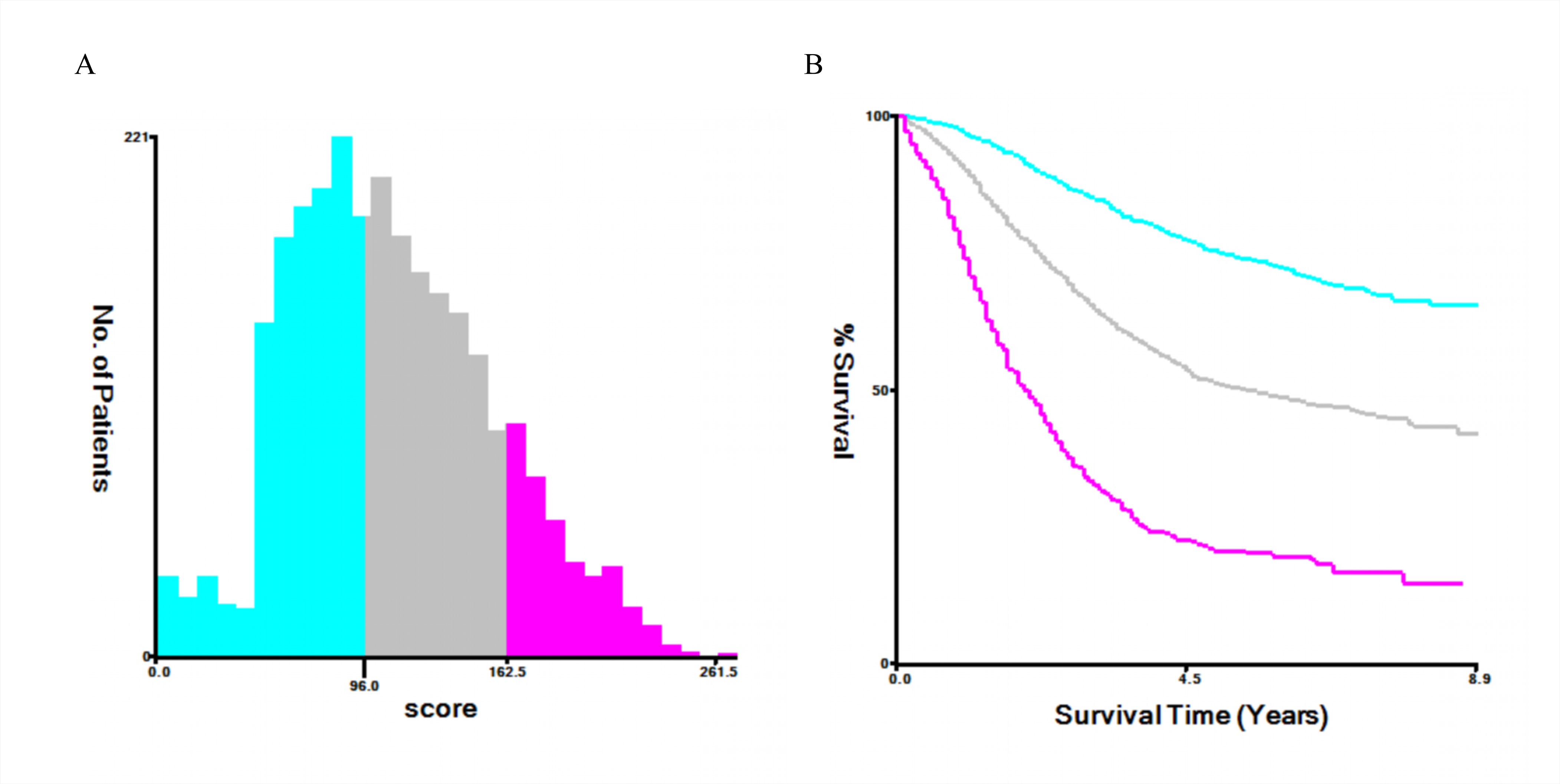

Supplement: Supplementary Figure 2 — Identification of the best cut-off point of total risk score in the validation cohort through X-tile software. [file Image_2.tif]
