# Supplementary material for: Construction and validation of a nomogram for predicting overall survival of patients with stage III/IV early−onset colorectal cancer
Source: Front Oncol. 2024 Apr 10;14:1332499. doi: 10.3389/fonc.2024.1332499 (PMC11040690; doi:10.3389/fonc.2024.1332499)
Supplement: Supplementary file 3 [file Table_1.docx]

**Table S1** Characteristics of patients stratified by AJCC stage.

| **Variables** | **Stage III (n=5197)** | **Stage IV (n=2933)** | **P value** |
| --- | --- | --- | --- |
| Age |  |  |  |
| Mean (SD) | 42.1 (6.19) | 42.3 (6.23) | 0.113 |
| Sex |  |  |  |
| Female | 2401 (46.2%) | 1356 (46.2%) | 0.996 |
| Male | 2796 (53.8%) | 1577 (53.8%) |  |
| Race/ethnicity |  |  |  |
| Hispanic | 923 (17.8%) | 507 (17.3%) | 0.034 |
| NHAIAN | 44 (0.8%) | 37 (1.3%) |  |
| NHAPI | 527 (10.1%) | 303 (10.3%) |  |
| NHB | 678 (13.0%) | 441 (15.0%) |  |
| NHW | 3025 (58.2%) | 1645 (56.1%) |  |
| Marital_status |  |  |  |
| Married | 3063 (58.9%) | 1655 (56.4%) | 0.029 |
| SDW | 2134 (41.1%) | 1278 (43.6%) |  |
| Primary_site |  |  |  |
| Left colon | 2308 (44.4%) | 1428 (48.7%) | <0.001 |
| Rectum | 1645 (31.7%) | 662 (22.6%) |  |
| Right colon | 1244 (23.9%) | 843 (28.7%) |  |
| Histologic_subtypes |  |  |  |
| Adenocarcinoma | 4700 (90.4%) | 2651 (90.4%) | 0.971 |
| MA/SRCC | 497 (9.6%) | 282 (9.6%) |  |
| Grade |  |  |  |
| I | 295 (5.7%) | 117 (4.0%) | <0.001 |
| II | 3738 (71.9%) | 1986 (67.7%) |  |
| III | 975 (18.8%) | 697 (23.8%) |  |
| IV | 189 (3.6%) | 133 (4.5%) |  |
| T_stage |  |  |  |
| T1 | 216 (4.2%) | 276 (9.4%) | <0.001 |
| T2 | 460 (8.9%) | 83 (2.8%) |  |
| T3 | 3522 (67.8%) | 1459 (49.7%) |  |
| T4 | 999 (19.2%) | 1115 (38.0%) |  |
| N_stage |  |  |  |
| N1 | 3369 (64.8%) | 1166 (39.8%) | <0.001 |
| N2 | 1828 (35.2%) | 1179 (40.2%) |  |
| N0 | 0 (0%) | 588 (20.0%) |  |

EO-CRC, early-onset colorectal cancer; SDW, separated, single, divorced, domestic partner or unmarried, widowed; NHW, Non-Hispanic White; NHB, Non-Hispanic Black; NHAPI,Non-Hispanic Asian or Pacific Islander; NHAIAN, Non-Hispanic American Indian/Alaska Native; MA, mucinous adenocarcinoma; SRCC, signet ring cell carcinoma; AJCC, American joint committee on cancer; T, Tumor; N, Node.
